# Identification and population genetic comparison of three ascidian species based on mtDNA sequences

**DOI:** 10.1002/ece3.6171

**Published:** 2020-03-10

**Authors:** Punit Bhattachan, Runyu Qiao, Bo Dong

**Affiliations:** ^1^ Key Laboratory of Marine Genetics and Breeding College of Marine Life Sciences Ocean University of China Qingdao China; ^2^ Laboratory for Marine Biology and Biotechnology Qingdao National Laboratory for Marine Science and Technology Qingdao China; ^3^ Institute of Evolution and Marine Biodiversity Ocean University of China Qingdao China

**Keywords:** ascidian, mtDNA sequence, population genetics, species identification

## Abstract

Ascidians are sessile marine chordate invertebrates found along seashores worldwide and are typically regarded as invasive organisms. Knowledge concerning their global genetic structure and subsequent invasive potential is limited. Here, we identified three ascidians—*Ciona robusta*, *Ciona savignyi*, and *Styela clava* from the northeast region of China using morphological characteristics and mitochondrial cytochrome *c* oxidase subunit I (*cox1*) as genetic marker. We additionally used phylogenetics to aid in the identification of these three species. The results of a population genetic analysis showed that among the three species, the level of haplotype diversity was particularly high within *C. savignyi*, and nucleotide diversity varied moderately. We divided the three species separately into native and invasive populations using 170 *cox1* sequences from global resources to explore population genetic structure and invasive potential. Although in the network analysis *Ciona* spp. formed haplogroups of native and invasive populations, some haplotypes were still shared. We found that the haplotypes did not cluster within the network of *S. clava*. Our AMOVA results also showed that *Ciona* spp. had a weak genetic structure, and less genetic differentiation was present in *S. clava*. These data suggest that there are extensive incursions of these three ascidians into different geographical regions. Global comparisons of ascidian populations will help in the understanding of their population genetic structure and invasive potential, hence providing important insights regarding conservation as well as management.

## Graphical abstract

Three ascidians—*Ciona robusta*, *Ciona savignyi*, and *Styela clava* were identified from the northeast region of China using morphological characteristics and mitochondrial cytochrome c oxidase subunit I (*cox1*) as genetic marker. *C. savignyi* has particularly high level of haplotype diversity and nucleotide diversity varied moderately. *Ciona* spp. had a weak genetic structure, and less genetic differentiation was present in *S. clava*. These data suggest that there are extensive incursions of these three ascidians into different geographical regions.

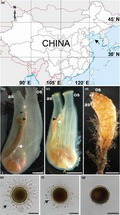

## 1 INTRODUCTION

The individuals of a species within in a population are not typically identical, and variation in DNA sequences and the resulting differences are regarded as genetic diversity, which are also known as polymorphisms (Ellegren & Galtier, 2016). This genetic diversity is important for the evolution of species as it allows for adaption to a particular environment (Väinölä & Johannesson, 2017). Mechanisms causing increased genetic diversity among species remain largely unknown (Leffler et al., 2012). Species in the wild with effectively large population sizes typically have high genetic diversity, as predicted by the neutral mutation hypothesis (Casillas & Barbadilla, 2017). By contrast, self‐fertilizing, inbred, and/or domesticated species have comparatively less genetic diversity (Cutter, Jovelin, & Dey, 2013). Recently, a correlation between life‐history traits and level of genetic diversity within species across phylogeny has been elucidated (Romiguier et al., 2014).

Ascidians, also known as sea squirts, are the most abundant class of the subphylum Tunicata and are distributed along shorelines worldwide (Shenkar & Swalla, 2011). They are sessile marine invertebrates belonging to the subphylum prochordata and are widely used as a model organism for developmental and evolutionary studies (Lemaire, 2011). Ascidians exhibit multiple morphological characteristics, from small colonial to colorful and large solitary forms. They are divided into three major well‐accepted orders, namely, Phlebobranchia, Aplousobranchia, and Stolidobranchia, based on the branchial sac morphology of the adults (Stolfi & Brown, 2015). However, within the phylogenetic tree, Phlebobranchia and Aplousobranchia show a closer relationship with Thaliaceae, which is also a Tunicata class different from Ascidiacea, whereas Stolidobranchia remains a distinct and monophyletic group (Delsuc et al., 2018). Over the course of several decades, the Ascidiacea have been shown to be an important class for ecological species because of their invasive potential (Zhan, Briski, Bock, Ghabooli, & MacIsaac, 2015) along with their ability to adapt to new environments (Hawes et al., 2019). Transportation of ascidians attached to ship hulls as fouling material and within the ballast water of ships has enabled them to invade new territories (Lambert, 2007). This phenomenon has major impacts on local marine biodiversity as well as aquaculture industries. Therefore, the Ascidiacea were recently considered as important model species for the study of nonindigenous species (NIS) worldwide (Zhan et al., 2015).

The mitochondrial genome is highly conserved across bilaterian phyla and typically contains 37 genes (Boore, 1999), with only 13 protein‐coding genes. Among them, *cox*1 is used as a DNA barcode for species identification (Hebert, Ratnasingham, & deWaard, 2003), genetic diversity patterns, and population genetic analysis (Moritz, Dowling, & Brown, 1987). In the recent decades, several Tunicata mitochondrial genomes have been sequenced (Gissi, Iannelli, & Pesole, 2004; Griggio et al., 2014; Singh et al., 2009; Yokobori, Oshima, & Wada, 2005; Yokobori, Watanabe, & Oshima, 2003). The sequencing data revealed that Tunicata species have high mutation rates compared with other animals (Griggio et al., 2014). Gene arrangements in the mitogenome of Tunicata are not conserved, suggesting high plasticity (Gissi et al., 2010). A mitogenomic approach has been used for cryptic species identification within *Ciona* (Iannelli, Pesole, Sordino, & Gissi, 2007), as well as for population genetic study for several ascidian species (Goldstien et al., 2011; Lopez‐Legentil, Turon, & Planes, 2006; Villalobos, Lambert, Shenkar, & Lopez‐Legentil, 2017; Zhan et al., 2012).

Here, we identified three ascidian species *Ciona robusta* (Hoshino & Tokioka, 1967), *Ciona savignyi* (Herdman, 1882), and *Styela clava* (Herdman, 1882) from northeast of China and examined their genetic diversity patterns. A comparative analysis of these species isolated from China with the samples elsewhere in the world using *cox*1 as a genetic marker was also performed to distinguish native from invasive ascidian populations.

## 2 MATERIALS AND METHODS

### 2.1 Animal collection

Adult animals were collected from the Rongcheng Bay area (37°55′N, 122°12′E China) and were maintained in the laboratory in seawater tanks with aeration and constant illumination. Ascidian species were identified morphologically. *C. robusta*, *C. savignyi*, and *S. clava* adults were dissected, and internal tissues were collected for DNA extraction and sequencing.

### 2.2 DNA extraction, cox1 amplification, and sequencing

Tissue sections were deposited into Eppendorf tubes containing digestion buffer and were incubated overnight at 50°C with shaking. In the following day, samples were treated with RNAase for 15 min at 37°C to remove total RNA, and the phenol‐chloroform extraction method was used for genomic DNA extraction. Around 500 µl of phenol‐chloroform was mixed and incubated at room temperature for 5 min with rocking. The mixture was then centrifuged for 10 min at room temperature. The upper aqueous layer was transferred to a fresh tube, and 1 ml ethanol was added for DNA precipitation. Tubes were centrifuged again for 10 min at room temperature, and the supernatant was discarded. DNA pallets were air dried and then resuspended in water. DNA quality was checked on a 1% agarose gel, and concentration was measured using a Nanodrop spectrophotometer. The primers for the amplification of *cox*1 from *C. robusta*, *C. savignyi*, and *S. clava* were designed according to published mitogenomes (Gissi et al., 2004; Griggio et al., 2014; Yokobori et al., 2003) as follows: (5′‐CATATAGTTTGAAACTATAAGATTC‐3′, 5′‐AGCCTTAAATACTGGTGAAG‐3′), (5′‐ATGTATAATTGATTAAATCGTTGG‐3′, 5′‐TCTTTTCATAACTGGAGATAC‐3′), and (5′‐ATGAGTTGAACTATTCGATGATTG‐3′, 5′‐CTTACTTAATATAAAAACTGGCCCT‐3′), respectively. PCR products were subcloned into a TA vector, and the correct clones were then sequenced.

### 2.3 Phylogenetic analysis

The *cox*1 sequences from three ascidian populations at different regions of the world (Table S1) were retrieved from the NCBI database to build multiple sequence alignments using ClustalW (Thompson, Higgins, & Gibson, 1994) hosted by MEGA7.0 (Kumar, Stecher, & Tamura, 2016). Only the unique haplotype datasets were used for the multiple sequence alignments (Table S1). Neighbor‐Joining (NJ) and maximum parsimony (MP) methods were employed to construct a phylogenetic tree with 1,000 bootstrap estimations in the default setting using MEGA7.0 (Kumar et al., 2016).The barcode region of the *cox*1 sequence (accession no. HM151268.1) of *Halocynthia roretzi* was used as an out‐group.

### 2.4 Genetic diversity, neutrality test, and population structure

Multiple sequence alignments of *cox1* from three ascidians were performed separately in ClustalW (Thompson et al., 1994) hosted by MEGA7.0 (Kumar et al., 2016) using default settings. Genetic diversity parameters, including haplotype number (H), haplotype diversity (Hd), nucleotide difference (K), mutation number per sequence (*θ*), number of segregating sites (S), and nucleotide diversity (Pi), were estimated using DnaSP software (Rozas, Sanchez‐DelBarrio, Messeguer, & Rozas, 2003). For the neutrality test, Tajima's *D* and Fu and Li's D* methods were used, with an additional evaluation using DnaSP software (Rozas et al., 2003). Relationships among the three ascidians *cox*1 haplotypes found globally, including those from China (Table S1), were determined using a median‐joining method in the network software (Bandelt, Forster, & Rohl, 1999). To infer the population structure and understand the connectivity between native and invasive ascidian populations, we performed molecular variance (AMOVA) analysis using *cox*1 haplotypes from samples available in the database (Table S1) as well as those in this study using the ARLEQUIN 3.11 software (Excoffier & Lischer, 2010) with 1,000 permutations.

## 3 RESULTS

### 3.1 Identification of three ascidians by morphological characteristics

**Figure 1:**
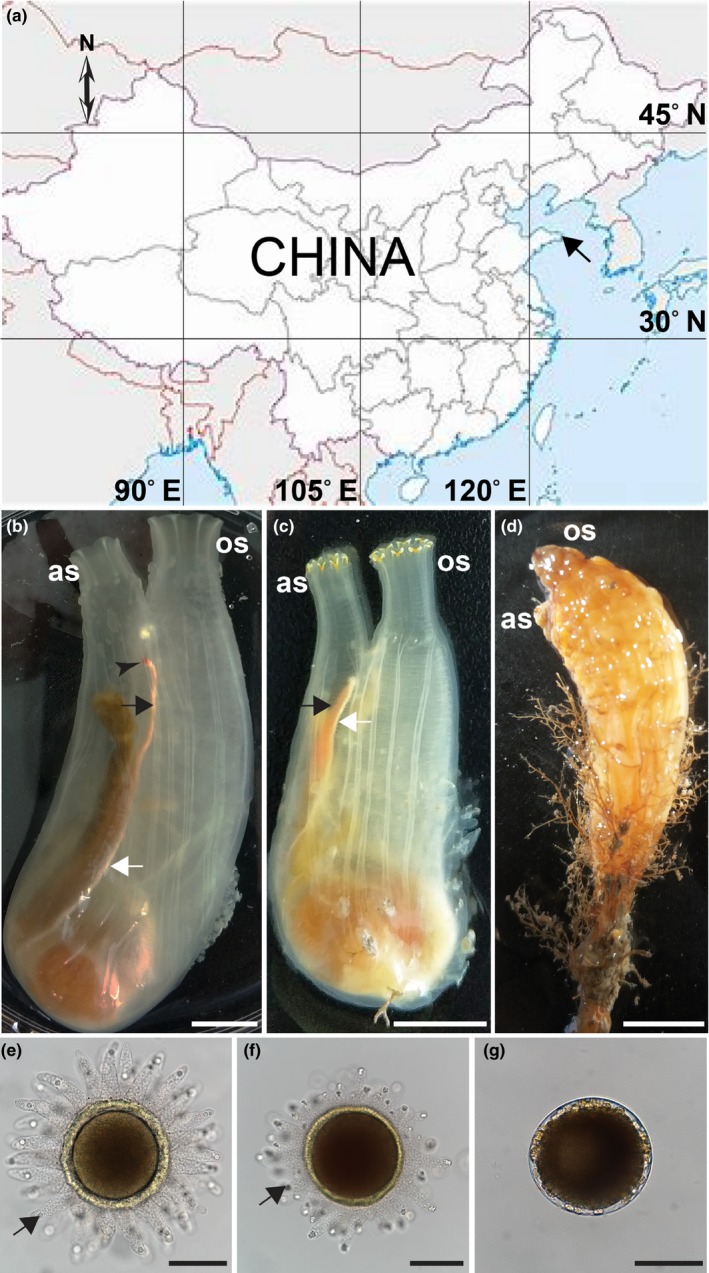
Morphological identification of three ascidian species. (a) Collection site of three ascidian samples (black arrow). (b) *C. robusta* adult with oral siphon (os), atrial siphon (as), sperm duct (white arrow), oviduct (black arrow), and red color at the tip of the sperm duct (arrowhead). (c) *C. savignyi* adult with oral siphon (os), atrial siphon (as), sperm duct (white arrow), and oviduct (black arrow). (d) Adult *S. clava* with oral siphon (os) and atrial siphon (as). Scale bar represents 1 cm. (e) The mature egg from *C. robusta* with long follicle cells (black arrow). (f) *C. savignyi* mature egg with short follicle cells (black arrow). (g) The mature egg of *S. clava* with no outer follicle cells. Scale bar represents 100 µm

All three ascidian samples were collected from the Rongcheng Bay area, which is a part of the Yellow Sea in Northeast China (black arrow, Figure 1a). The overall adult morphology between *Ciona* spp. and *S. clava* consisted of a vase‐like shape. The tunic in *Ciona* spp. was soft and semitransparent, whereas that in *S. clava* was hard and dark brown (Figure 1b–d). Consequently, the gonoducts were clearly visible by naked eye in *Ciona* spp. (arrows in Figure 1b,c), but were not visible in *S. clava* adults (Figure 1d). All three species were solitary, sessile, and attached on a submerged surface. In addition, there were other distinguishing features between *Ciona* spp. as the outer tunic of *C. robusta* was comparatively thicker and rougher than that of *C. savignyi*. Red coloration was present at the tip of the sperm duct in *C. robusta* (arrowhead, in Figure 1b), which was not observed in *C. savignyi*. We also examined the egg morphology to identify and distinguish the three species. The eggs of *Ciona* spp. had follicle cells on the outer covering, and the length of the follicle cells in *C. robusta* was longer (arrow, Figure 1e) than that in *C. savignyi* (arrow, Figure 1f). By contrast, no outer follicle cells were present on the eggs of *S. clava* (Figure 1g).

### 3.2 Molecular identification of three ascidians by the cox1 gene

We cloned the full length of the *cox1* gene from genomic DNA of 50 individuals of the three ascidian species. Each resulting sequence was subjected to BLASTN (Altschul, Gish, Miller, Myers, & Lipman, 1990), with the results indicating that these sequences belonged to the three respective ascidian species. The open reading frames of the *cox1* sequence from three species were variable. We identified a deletion polymorphism of *cox1* in *C*. *savignyi*, but not in *C. robusta* and *S. clava*. For instance, only a single 1,560 and 1,543 bp‐length of *cox1* sequence was identified in *C. robusta* and *S. clava*, respectively, whereas two different lengths of *cox1* (1,545 and 1,548 bp) were identified in *C*. *savignyi*. All these sequences were deposited in the NCBI database, and the accession numbers are listed in Table S1.

**Figure 2:**
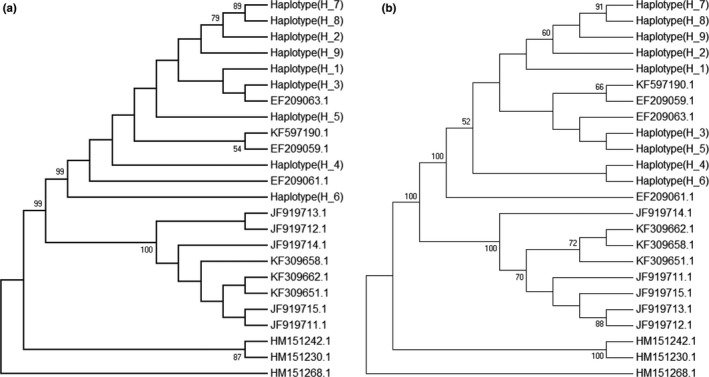
Phylogenetic trees of *C. robusta* population constructed using *cox*1 bar code fragment. The haplotypes (H_1 to H_9) are presented from this study. (a) Population phylogeny of 23 haplotypes using neighbor‐joining (NJ) method. (b) Population phylogeny of 23 haplotypes using maximum parsimony (MP) method. Bootstrap values are presented at the nodes with more than 50% value

**Figure 3:**
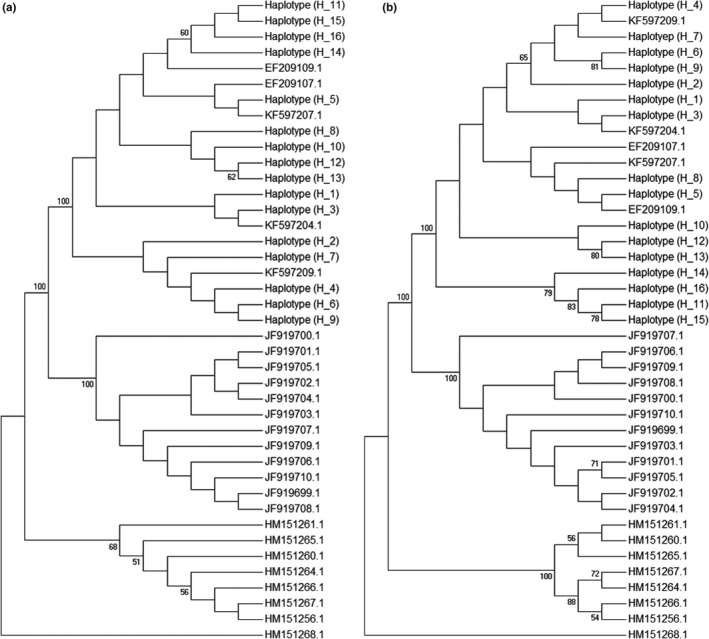
Phylogenetic trees of *C. savignyi* population constructed using *cox*1 bar code fragment. The haplotypes (H_1 to H_16) are used from this study. (a) Neighbor‐joining (NJ) method was utilized to construct population phylogeny from 40 haplotypes. (b) Population phylogeny of 40 haplotypes using maximum parsimony (MP) method. More than 50% bootstrap values are presented at the nodes

**Figure 4:**
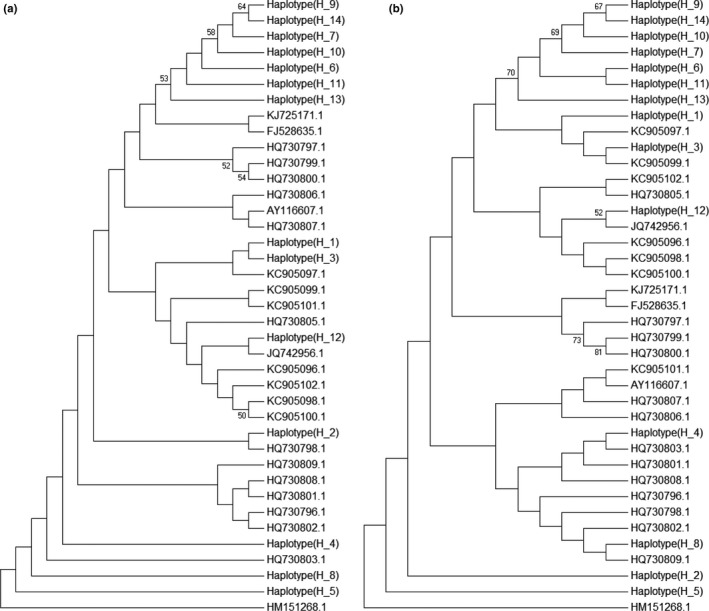
Phylogenetic trees of *S. clava* population constructed using *cox*1 bar code fragment. The haplotypes (H_1 to H_14) are from this study. (a) Population phylogeny of 38 haplotypes constructed by neighbor‐joining (NJ) method. (b) Maximum parsimony (MP) method was applied to construct population phylogeny from 38 haplotypes. Bootstrap values of more than 50% values are presented at the nodes

We also retrieved the *cox1* barcode sequences from the NCBI database (Table S1). Only the *cox*1 barcode regions of unique haplotypes were used for multiple sequence alignments and phylogenetic tree construction. The resulting phylogenetic trees allowed us to delineate different haplotypes among all of the samples. In the *C. robusta* tree, we found that the haplotypes (H_1 to H_9) from China did not form a single clade in either NJ (Figure 2a) or MP (Figure 2b) trees, but rather clustered with some haplotypes from individuals originating from Korea and the USA. Similarly, the haplotypes (H_1 to H_16) of *C. savignyi* from China did not cluster in a single clade in either NJ (Figure 3a) or MP (Figure 3b) trees. Instead, they grouped with other haplotypes from Korea and the USA. In addition, NJ (Figure 4a) and MP (Figure 4b) trees did not resolve the haplotypes (H_1 to H_14) of *S. clava* from China into a single clade either. Conversely, they formed a cluster with some haplotypes from New Zealand and the USA, which were invasive populations.

### 3.3 Haplotype identification and diversity of the cox1 gene marker

We used the *cox1* gene for molecular diversity analysis. Nine haplotypes were identified among 14 *C. robusta* samples, 14 haplotypes among 19 *S. clava* samples, and 16 haplotypes among 17 *C. savignyi* samples (Table 1). The results of the comparative analysis using different genetic diversity parameters also revealed that *C. savignyi* was diverse compared with *C. robusta* and *S. clava*. The haplotype diversity (Hd) was comparatively higher in *C. savignyi* (0.993 + 0.038) than that in *C. robusta* (0.912 + 0.059), and *S. clava* (0.947 + 0.038). Similarly, the detected average number of nucleotide difference (K) in *C. savignyi* (20.618) was higher than that in *C. robusta* (8.143) and *S. clava* (11.550). Nucleotide diversity (Pi) and average number of mutations (θ) were also relatively higher in *C. savignyi* (0.02630, 0.05061) compared with *C. robusta* (0.01094, 0.01811) and *S. clava* (0.01919, 0.03097), respectively (Table 1).

**Table 1:** Summary statistics of genetic diversity from three ascidian species

Species	Sample size	No of haplotype (H)	Haplotype diversity (Hd) + *SD*	Nucleotide difference (K)	No of mutation per site (*θ*)	No of segregating sites (S)	Nucleotide diversity (Pi)
*Ciona robusta*	14	9	0.912 ± 0.059	8.143	0.01811	41	0.01094
*Ciona savignyi*	17	16	0.993 ± 0.023	20.618	0.05061	122	0.02630
*Styela clava*	19	14	0.947 + 0.038	11.550	0.03097	60	0.01919

Next, we carried out the neutrality test. The Tajima's *D* values were negative for all three species (Table 2), but these values were significant only in *C. savignyi* population (*p* < .05), indicating that there was a excess of low‐frequency polymorphisms, and the *C. savignyi* population was expanding. However, in the *C. robusta*/*S. clava* populations, the values were not statistically significant, indicating that these two species populations did not deviate from the neutral expectations. Similarly, for Fu and Li's D* statistic, negative values were observed in all three species (Table 2). The values from *C. robusta* and *C. savignyi* were statistically significant, whereas those from *S. clava* were not (*p* > .10). The results from these two analytical approaches indicate that the population of *C. savignyi* is undergoing positive selection and expansion.

**Table 2:** Test for neutrality from three ascidian species

Species	Tajima's *D* test	Significance (*p*)	Fu and Li's D* statistic	Significance (*p*)
*Ciona robusta*	−1.67103	>.05	−2.28224	<.50*
*Ciona savignyi*	−1.92716	<.05*	−2.40874	<.05*
*Styela clava*	−1.47586	>.10	−1.98094	>.10

### 3.4 Connectivity between native and non‐native ascidians

**Figure 5:**
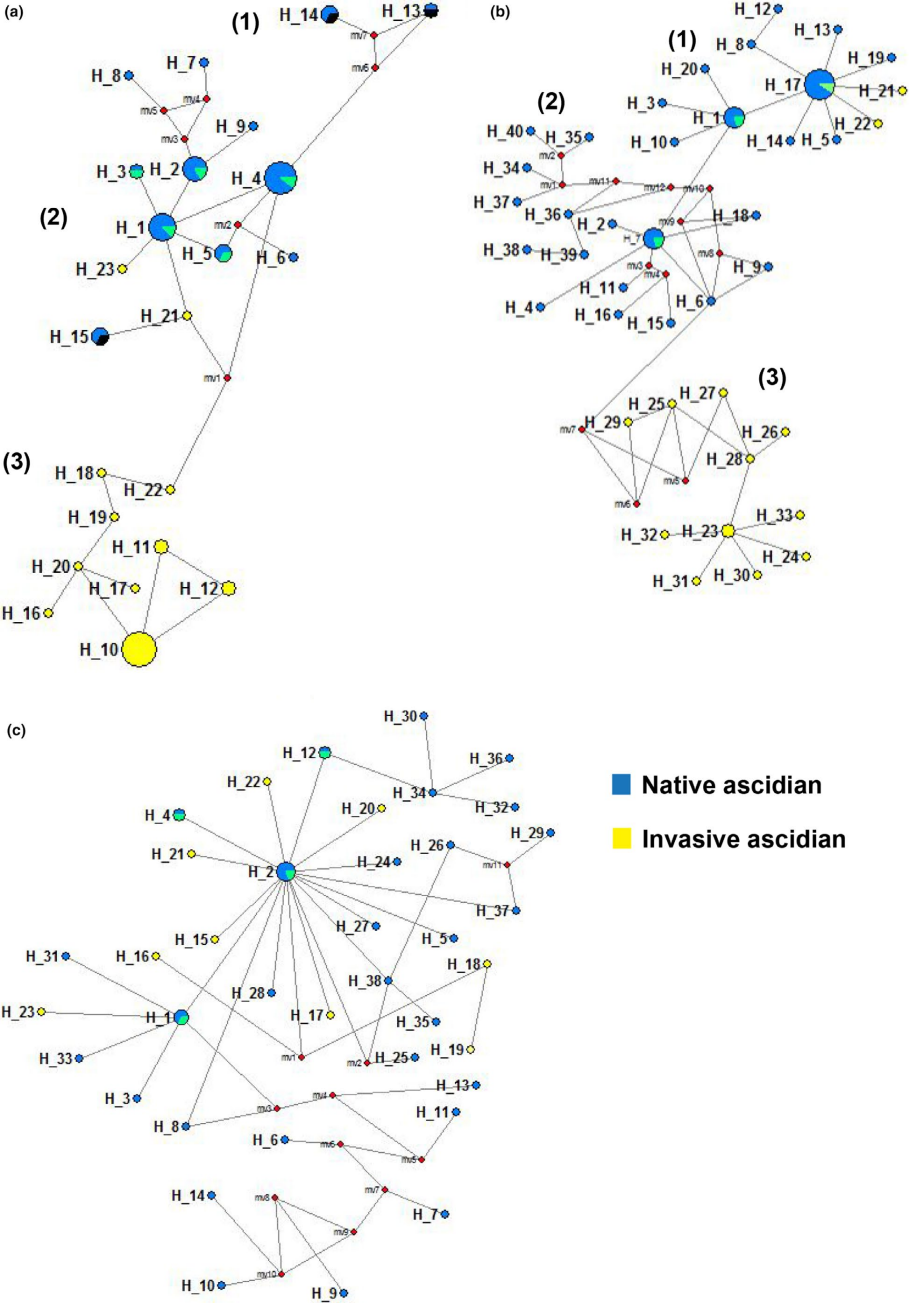
Network analysis of three ascidian species from 170 *cox1* sequences. The size of nodes is proportional to the haplotype frequency. The distance between nodes is proportional to the mutation. The blue color indicated the native haplotypes, yellow color showed the invasive haplotypes, whereas red diamonds indicated median vectors. Two different colors hinted admixture. (a) Network of *C. robusta* from *cox*1 haplotypes with 1, 2, and 3 haplogroups. (b) Network of *C. savignyi* from *cox*1 haplotypes with 1, 2, and 3 haplogroups. (c) Network of *S. clava* from *cox*1 haplotypes without haplogroups

We divided the three ascidian species populations into native and invasive groups, with populations located within eastern Asian countries‐like China, Japan, and Korea being considered as native groups. Since these species are believed to have originated from this region (Lambert & Lambert, 1998), while the rest of the populations from other regions were grouped as invasive populations. Network analysis revealed that there were three haplogroups (1, 2, and 3) in *C. robusta* (Figure 5a) and *C. savignyi* (Figure 5b), respectively. No haplogroups were found for *S. clava* (Figure 5c). In the *C. robusta* network, we found native populations in haplogroup 1, and haplogroup 3 consisted of invasive populations. On the other hand, haplogroup 2 was comprised mainly of native populations, including those from China, but few haplotypes were shared from invasive populations as well. Haplogroups 1 and 2 were connected with haplogroup 3 (Figure 5a). Similarly, in the *C. savignyi* network (Figure 5b), we found native populations in haplogroup 1, but haplogroup 2 was entirely composed of only native populations, and haplogroup 3 consisted only of invasive populations. By contrast, there were no haplogroups present in the *S. clava* network, and all haplotypes from both native and invasive populations, including those from China, were connected to each other (Figure 5c).

We also performed hierarchical AMOVA using *cox*1 haplotypes from both native and invasive populations of three ascidian species. There was no clear structure between native and invasive populations in *C. robusta* (11.94%, CT = 0.11941) and *C. savignyi* (6.58%, CT = 0.06582), but these values were not statistically significant (Table 3). In addition, we recorded a negative value for *S. clava* (−8.01%, CT = −0.08014), indicating that there was no population differentiation (Table 3). By contrast, among populations of *C. robusta* (66.99%, SC = 0.78928), *C. savignyi* (70.64%, SC = 0.75622), and *S. clava* (30.65%, SC = 0.28373), there were significant variations, with the highest level of variation appearing in *C. savignyi* (Table 3). Surprisingly, within these variations, the highest value was recorded for *S. clava* (77.37%, ST = 0.22632), followed by *C. savignyi* (22.77%, ST = 0.77226) and *C. robusta* (21.07%, ST = 0.76671; Table 3).

**Table 3:** AMOVA of three ascidian populations by mitochondrial *cox1* sequences

Species	Source of variance	*df*	Sum of squares	Variance components	Percentage of variation	Fixation indices	*p* Values
*Ciona robusta* (*n* = 66)	*Between native and invasive ascidians*						
	Among group	1	1664.677	15.24744	11.94	CT = 0.11941	.30205 ± .01599
	Among population within group	4	3,479.895	85.53321	66.99	SC = 0.78928	.00000 ± .00000
	Within populations	60	1614.337	26.90561	21.07	ST = 0.76071	.00000 ± .00000
*Ciona savignyi* (*n* = 59)	*Between native and invasive ascidians*						
	Among group	1	1551.825	10.18461	6.58	CT = 0.06582	.28446 ± .01465
	Among population within group	3	3,745.571	109.31788	70.64	SC = 0.75622	.00000 ± .00000
	Within populations	54	1903.003	35.24079	22.77	ST = 0.77226	.00000 ± .00000
*Styela clava* (*n* = 45)	*Between native and invasive ascidians*						
	Among group	1	8.899	−0.37431	−8.01	CT=−0.08014	.00684 ± .00231
	Among population within group	5	52.702	1.43135	30.65	SC = 0.28373	.01173 ± .00437
	Within populations	38	137.310	3.61343	77.37	ST = 0.22632	.00684 ± .00231

## 4 DISCUSSION

In the present study, we identified three ascidian species from the Northeast China using both morphological characteristics and genetic marker analysis. The tunic of *Ciona* spp. is soft and semitransparent, whereas that of *S. clava* is relatively rough and opaque. Since the tunic is mainly composed of a cellulose‐like material resembling that of plants (Nakashima, Yamada, Satou, Azuma, & Satoh, 2004), we assume that tunic composition varies among different species. In addition, *Ciona* spp. absorb more water, as demonstrated by dry tunic weight (Tarallo, Yagi, Oikawa, Agnisola, & D'Onofrio, 2016), and potentially as a result, this organ became semitransparent in nature. Furthermore, *C. robusta* is comparatively larger in size than *C. savignyi*. Recently, it was also revealed that the morpho‐physiological properties play an essential role in the control of size between these two ascidians (Tarallo et al., 2016). Hence, we can use these characters to distinguish between them. It is also interesting to note that there is a red coloration at the tip of the sperm duct in *C. robusta*, which is absent in *C. savignyi* (Lee & Shin, 2014). The evolutionary and functional property of this pigmentation is not yet known. Strikingly, egg morphology also varies among these three species. For instance, long follicle cells are present on the outer covering of *C. robusta* eggs, comparatively shorter follicular cells overlay *C. savignyi* eggs, and no outer follicle cells are present on *S. clava* eggs. Generally, the ascidian egg consists of two layers of follicle cells, with a vitelline coat next to the egg membrane and several test cells between them (Satoh, 2014). These outer follicle cells are vacuolated and elongated and are speculated to provide buoyancy to eggs in seawater (Kessel, 1967). This may help ascidian eggs disperse by the water current and thereby be transported to distant places. Follicle cells are also the first contact of sperm entry, and it is widely known that they function to prevent self‐fertilization via a chemical reaction (Pinto, Desantis, Marino, & Usui, 1995). Long follicle cells might have enabled a higher dispersal rate of *C. robusta*. This characteristic might also inhibit more self‐fertilization in comparison to *C. savignyi* and *S. clava*.

The genetic marker *cox*1 has been widely used for identification and characterization of genetic diversity (Hebert & Gregory, 2005). On the basis of barcode region of the *cox*1 gene from these three ascidian species as well as other available sequences in the databases, we constructed the phylogenetic trees to infer their identification, which showed that the *Ciona* spp. from China was closely related to native populations, mostly from Korea to Japan. This result indicates that the *Ciona* spp. samples collected here from China are indeed native ascidians, and these were not introduced from other geographical areas. However, *S. clava* formed a clade with invasive populations. We also found that some haplotypes from invasive populations formed a cluster with native populations. This result indicates that there was incursion of native and invasive ascidian populations to different parts of the world. A similar phylogenetic method was used for ascidian identification in other geographical regions as well (Iyappan, Ananthan, & Sathishkumar, 2016; Jaffarali, Akram, & Arshan, 2018; Lee & Shin, 2014; Smith, Cahill, & Fidler, 2010).

The ascidians are marine organisms with a relatively high level of genetic diversity (Leffler et al., 2012), and there exist differences in levels of genetic diversity among the ascidians themselves (Tsagkogeorga, Cahais, & Galtier, 2012). How these differing levels of genetic diversity are maintained remains unknown. Our current analyses confirmed that these ascidians have a high level of genetic diversity, with *C. savignyi* exhibiting a comparatively high level of genetic diversity at the molecular level. One possible explanation might be that *C. savignyi* has a large effective population size, with differing life‐history traits compared to *C. robusta* and *S. clava*. Of note, a previous genome‐wide study also revealed that *C. savignyi* exhibited the highest level of genetic diversity (Small, Brudno, Hill, & Sidow, 2007). Other comparative studies on ascidians also confirmed that they have different evolutionary rates (Berna & Alvarez‐Valin, 2014). This could be another reason causing the different levels of genetic diversity among these three species. In addition, the neutrality tests showed that *C*. *robusta* and *S. clava* are undergoing neutral evolution, and *C. savignyi* is experiencing population expansion and positive selection. This also explains why *C. savignyi* exhibits a higher level of genetic diversity compared with *C. robusta* and *S. clava*. Given the widespread distribution of ascidians, it is possible to exhibit high genetic diversity across populations. This kind of observation is also seen in a wide range of other organisms (Ellegren & Galtier, 2016).

Another important characteristic feature of ascidians is their invasive potential. Some ascidian species are dispersed to different geographical or ecological niches because of both anthropogenic and natural causes and are hereby considered as invasive species (Lambert & Lambert, 1998). We compared the global *cox*1 haplotypes of these three ascidians to understand their connectivity and population genetic structure. Global haplotypes were divided into native and invasive populations. The network analysis indicated that *Ciona* spp. formed haplogroups with separate native and invasive populations, although some haplotypes were shared. However, in the network of *S. clava*, there was no such haplogroup formation as all of its haplotypes were interconnected, suggesting extensive incursion for this species in different geographical areas. A previous global study of *S. clava* also suggested its extensive incursion, in which it was categorized as invasive species (Goldstien et al., 2011). In addition, a regional study of this species indicated the multiple sources of incursions (Goldstien, Schiel, & Gemmell, 2010). The results of the hierarchical AMOVA analysis of the three species here were also consistent with the network analysis. We found a weak population genetic structure in *Ciona* spp. and less genetic differentiation in *S. clava* populations. An occasional gene flow between native and invasive populations of ascidians might have occurred previously, most likely via ship transport. We clearly show that the *C*. *robusta* and *S. clava* invasive potential is attributed to the neutral genetic diversity, whereas the invasive potential of *C. savignyi* might not be due to neutral evolution, but rather by population expansion and positive selection. Previous work indicated that a neutral force plays a role in the biological invasion and subsequent structuring of a population (Daleo, Alberti, & Iribarne, 2009), but equally natural selection within biological invasion was also well characterized (Lee, 2002). It is worth noting that our analysis was based on the small sample size, because of the fewer collection sites. Increase of collection sites and sample sizes could be more accurate for the population genetic evaluation, but would not change the conclusion. Our current study reveals a global relationship between native and invasive populations and has implications in understanding the invasive potential of these three species. Thus, our work provides approaches useful for risk evaluation and management of invasive species.

## CONFLICT OF INTEREST

The authors report no conflict of interest.

## AUTHOR CONTRIBUTIONS

**Punit Bhattachan**: Conceptualization (equal); data curation (equal); formal analysis (equal); visualization (equal); writing‐original draft (equal); writing‐review and editing (equal). **Runyu Qiao**: Data curation (equal); formal analysis (equal); validation (equal). **Bo Dong**: Conceptualization (equal); formal analysis (equal); funding acquisition (lead); project administration (lead); resources (lead); supervision (lead); writing‐review and editing (lead).

## Supporting information

Table S1

## Data Availability

All DNA sequences were deposited in NCBI: Genbank accessions MK012337–MK012341 and MN890028–MN890047.
