# Supplementary material for: Identification and population genetic comparison of three ascidian species based on mtDNA sequences
Source: Ecol Evol. 2020 Mar 10;10(8):3758–68. doi: 10.1002/ece3.6171 (PMC7160174; doi:10.1002/ece3.6171)
Supplement: Supplementary file 1 — Table S1 [file ECE3-10-3758-s001.docx]

**Supplemental materials**

**Table S1 List of *cox*1 sequences from three ascidian species used in this study**

(a) *Ciona robusta*

| SN | NCBI accession number | Geographical region and haplotypes | SN | NCBI accession number | Geographical region  and haplotypes |
| --- | --- | --- | --- | --- | --- |
| 1 | MK012337 | China (Haplotype_1, n=2) | 38 | KF309593.1 | Spain Haplotype_10) |
| 2 | MK012338 | China (Haplotype_2, n=2) | 39 | KF309591.1 | Spain (Haplotype_10) |
| 3 | MK012339 | China (Haplotype_3, n=1) | 40 | KF309587.1 | Spain (Haplotype_10) |
| 4 | MK012340 | China (Haplotype_4, n=4) | 41 | KF309580.1 | Spain (Haplotype_10) |
| 5 | MK012341 | China (Haplotype_5, n=1) | 42 | KF309578.1 | Spain (Haplotype_10) |
| 6 | MN890028 | China (Haplotype_6, n=1) | 43 | KF309570.1 | Spain (Haplotype_10) |
| 7 | MN890029 | China (Haplotype_7, n=1) | 44 | KF309554.1 | Spain (Haplotype_12) |
| 8 | MN890030 | China (Haplotype_8, n=1) | 45 | KF309532.1 | Spain (Haplotype_10) |
| 9 | MN890031 | China (Haplotype_9, n=1) | 46 | EF209056.1 | USA (Haplotype_15) |
| 10 | KF597182.1 | Korea (Haplotype_1) | 47 | EF209057.1 | USA (Haplotype_1) |
| 11 | KF597183.1 | Korea (Haplotype_4) | 48 | EF209058.1 | USA (Haplotype_1) |
| 12 | KF597184.1 | Korea (Haplotype_2) | 49 | EF209059.1 | USA (Haplotype_21) |
| 13 | KF597185.1 | Korea (Haplotype_1) | 50 | EF209060.1 | USA (Haplotype_1) |
| 14 | KF597186.1 | Korea (Haplotype_4) | 51 | EF209061.1 | USA (Haplotype_22) |
| 15 | KF597187.1 | Korea (Haplotype_4) | 52 | EF209062.1 | USA (Haplotype_2) |
| 16 | KF597188.1 | Korea (Haplotype_4) | 53 | EF209063.1 | USA (Haplotype_23) |
| 17 | KF597189.1 | Korea (Haplotype_4) | 54 | EF209064.1 | USA (Haplotype_3) |
| 18 | KF597190.1 | Korea (Haplotype_15) | 55 | EF209065.1 | USA (Haplotype_5) |
| 19 | KF597191.1 | Korea (Haplotype_15) | 56 | EF209061.1 | USA (Haplotype_22) |
| 20 | KF597192.1 | Korea (Haplotype_4) | 57 | EF209066.1 | USA (Haplotype_2) |
| 21 | KF597193.1 | Korea (Haplotype_1) | 58 | JF919711.1 | New Zealand (Haplotype_21) |
| 22 | KF597194.1 | Korea (Haplotype_2) | 59 | JF919712.1 | New Zealand  (Haplotype_19) |
| 23 | KF597195.1 | Korea (Haplotype_5) | 60 | JF919713.1 | New Zealand  (Haplotype_18) |
| 24 | HM151231.1 | Japan (Haplotype_13) | 61 | JF919714.1 | New Zealand  (Haplotype_17) |
| 25 | HM151230.1 | Japan (Haplotype_14) |  |  |  |
| 26 | HM151249.1 | Japan (Haplotype_14) |  |  |  |
| 27 | HM151236.1 | Japan (Haplotype_14) |  |  |  |
| 28 | HM151242.1 | UK (Haplotype_13) |  |  |  |
| 29 | KF309662.1 | Spain (Haplotype_10) |  |  |  |
| 30 | KF309658.1 | Spain (Haplotype_11) |  |  |  |
| 31 | KF309651.1 | Spain (Haplotype_12) |  |  |  |
| 32 | KF309628.1 | Spain (Haplotype_10) |  |  |  |
| 33 | KF309614.1 | Spain (Haplotype_10) |  |  |  |
| 34 | KF309613.1 | Spain (Haplotype_11) |  |  |  |
| 35 | KF309604.1 | Spain (Haplotype_10) |  |  |  |
| 36 | KF309603.1 | Spain (Haplotype_10) |  |  |  |
| 37 | KF309602.1 | Spain (Haplotype_10) |  |  |  |

(b) *Ciona savignyi*

| SN | NCBI accession number | Geographical location  and haplotypes | SN | NCBI accession number | Geographical location  and haplotypes |
| --- | --- | --- | --- | --- | --- |
| 1 | MK012342 | China (Haplotype_1, n=1) | 32 | KF597205.1 | Korea (Haplotype_10) |
| 2 | MK012343 | China (Haplotype_2, n=1) | 33 | KF597204.1 | Korea (Haplotype_13) |
| 3 | MK012344 | China (Haplotype_3, n=1) | 34 | KF597203.1 | Korea (Haplotype_10) |
| 4 | MK012345 | China (Haplotype_4, n=1) | 35 | KF597202.1 | Korea (Haplotype_1) |
| 5 | MK012346 | China (Haplotype_5, n=1) | 36 | KF597201.1 | Korea (Haplotype_1) |
| 6 | MK012347 | China (Haplotype_6, n=1) | 37 | KF597200.1 | Korea (Haplotype_10) |
| 7 | MK012348 | China (Haplotype_7, n=2) | 38 | KF597198.1 | Korea (Haplotype_10) |
| 8 | MK012349 | China (Haplotype_8, n=1) | 39 | KF597199.1 | Korea (Haplotype_10) |
| 9 | MK012350 | China (Haplotype_9, n=1) | 40 | KF597197.1 | Korea (Haplotype_10) |
| 10 | MN890032 | China (Haplotype_10, n=1) | 41 | KF597196.1 | Korea (Haplotype_10) |
| 11 | MN890033 | China (Haplotype_11, n=1) | 42 | EF209106.1 | USA (Haplotype_10) |
| 12 | MN890034 | China (Haplotype_12, n=1) | 43 | EF209107.1 | USA (Haplotype_14) |
| 13 | MN890035 | China (Haplotype_13, n=1) | 44 | EF209108.1 | USA (Haplotype_7) |
| 14 | MN890036 | China (Haplotype_14, n=1) | 45 | EF209109.1 | USA (Haplotype_15) |
| 15 | MN890037 | China (Haplotype_15, n=1) | 46 | EF209110.1 | USA (Haplotype_1) |
| 16 | MN890038 | China (Haplotype_16, n=1) | 47 | JF919699.1 | New Zealand (Haplotype_16) |
| 17 | HM151267.1 | Japan (Haplotype_28) | 48 | JF919700.1 | New Zealand (Haplotype_17) |
| 18 | HM151266.1 | Japan (Haplotype_29) | 49 | JF919701.1 | New Zealand (Haplotype_18) |
| 19 | HM151265.1 | Japan (Haplotype_30) | 50 | JF919702.1 | New Zealand (Haplotype_19) |
| 20 | HM151264.1 | Japan (Haplotype_31) | 51 | JF919703.1 | New Zealand (Haplotype_20) |
| 21 | HM151261.1 | Japan (Haplotype_32) | 52 | JF919704.1 | New Zealand (Haplotype_21) |
| 22 | HM151260.1 | Japan (Haplotype_33) | 53 | JF919705.1 | New Zealand (Haplotype_22) |
| 23 | HM151256.1 | Japan (Haplotype_34) | 54 | JF919706.1 | New Zealand (Haplotype_23) |
| 24 | KF597208.1 | Korea (Haplotype_10) | 55 | JF919707.1 | New Zealand (Haplotype_24) |
| 25 | KF597209.1 | Korea (Haplotype_11) | 56 | JF919708.1 | New Zealand (Haplotype_25) |
| 26 | KF597210.1 | Korea (Haplotype_7) | 57 | JF919709.1 | New Zealand (Haplotype_26) |
| 27 | KF597211.1 | Korea (Haplotype_1) | 58 | JF919710.1 | New Zealand (Haplotype_27) |
| 28 | KF597212.1 | Korea (Haplotype_10) |  |  |  |
| 29 | KF597213.1 | Korea (Haplotype_7) |  |  |  |
| 30 | KF597207.1 | Korea (Haplotype_12) |  |  |  |
| 31 | KF597206.1 | Korea (Haplotype_10) |  |  |  |

(c) *Styela clava*

| SN | NCBI accession number | Geographical location and haplotypes |
| --- | --- | --- |
| 1 | MK012351 | China (Haplotype_1, n=3) |
| 2 | MK012352 | China (Haplotype_2, n=4) |
| 3 | MK012353 | China (Haplotype_3, n=1) |
| 4 | MK012354 | China (Haplotype_4, n=1) |
| 5 | MK012355 | China (Haplotype_5, n=1) |
| 6 | MN890039 | China (Haplotype_6, n=1) |
| 7 | MN890040 | China (Haplotype_7, n=1) |
| 8 | MN890041 | China (Haplotype_8, n=1) |
| 9 | MN890042 | China (Haplotype_9, n=1) |
| 10 | MN890043 | China (Haplotype_10, n=1) |
| 11 | MN890044 | China (Haplotype_11, n=1) |
| 12 | MN890045 | China (Haplotype_12, n=1) |
| 13 | MN890046 | China (Haplotype_13, n=1) |
| 14 | MN890047 | China (Haplotype_14, n=1) |
| 15 | HQ730796.1 | Japan (Haplotype_6) |
| 16 | HQ730797.1 | Japan (Haplotype_7) |
| 17 | HQ730798.1 | Japan (Haplotype_8) |
| 18 | HQ730799.1 | Japan (Haplotype_9) |
| 19 | HQ730800.1 | Japan (Haplotype_10) |
| 20 | HQ730801.1 | Japan (Haplotype_11) |
| 21 | HQ730802.1 | Japan (Haplotype_12) |
| 22 | HQ730803.1 | Japan (Haplotype_13) |
| 23 | JQ742956.1 | Korea (Haplotype_20) |
| 24 | KJ725171.1 | India (Haplotype_19) |
| 25 | AY116607.1 | France (Haplotype_29) |
| 26 | FJ528636.1 | Spain (Haplotype_2) |
| 27 | FJ528635.1 | Spain (Haplotype_28) |
| 28 | HQ730795.1 | USA (Haplotype_4) |
| 29 | HQ730805.1 | USA (Haplotype_14) |
| 30 | HQ730806.1 | USA (Haplotype_15) |
| 31 | HQ730807.1 | USA (Haplotype_16) |
| 32 | HQ730808.1 | USA (Haplotype_17) |
| 33 | HQ730809.1 | USA (Haplotype_18) |
| 34 | KC905096.1 | New Zealand (Haplotype_21) |
| 35 | KC905097.1 | New Zealand (Haplotype_22) |
| 36 | KC905098.1 | New Zealand (Haplotype_23) |
| 37 | KC905099.1 | New Zealand (Haplotype_24) |
| 38 | KC905100.1 | New Zealand (Haplotype_25) |
| 39 | KC905101.1 | New Zealand (Haplotype_26) |
| 40 | KC905102.1 | New Zealand (Haplotype_27) |
